# Metformin overdose causes platelet mitochondrial dysfunction in humans

**DOI:** 10.1186/cc11663

**Published:** 2012-10-03

**Authors:** Alessandro Protti, Anna Lecchi, Francesco Fortunato, Andrea Artoni, Noemi Greppi, Sarah Vecchio, Gigliola Fagiolari, Maurizio Moggio, Giacomo Pietro Comi, Giovanni Mistraletti, Barbara Lanticina, Loredana Faraldi, Luciano Gattinoni

**Affiliations:** 1 Dipartimento di Anestesia, Rianimazione (Intensiva e Sub-Intensiva) e Terapia del Dolore, Fondazione IRCCS Ca' Granda - Ospedale Maggiore Policlinico, Università degli Studi di Milano, via F. Sforza 35, 20122 Milan, Italy; 2 Centro Emofilia e Trombosi Angelo Bianchi Bonomi, Fondazione IRCCS Ca' Granda - Ospedale Maggiore Policlinico, via F. Sforza 35, 20122 Milan, Italy; 3 Centro Dino Ferrari - Dipartimento di Scienze Neurologiche, Fondazione IRCCS Ca' Granda - Ospedale Maggiore Policlinico, Università degli Studi di Milano, via F. Sforza 35, 20122 Milan, Italy; 4 Centro Trasfusionale e di Immunoematologia, Dipartimento di Medicina Rigenerativa, Fondazione IRCCS Ca' Granda - Ospedale Maggiore Policlinico, via F. Sforza 35, 20122 Milan, Italy; 5 Centro Nazionale di Informazione Tossicologica - Centro Antiveleni, Fondazione IRCCS Salvatore Maugeri, via S. Maugeri 10/10A, 27100 Pavia, Italy; 6 U.O. Anestesia e Rianimazione, A.O. San Paolo, Università degli Studi di Milano, via A. Di Rudiní 8, 20142 Milan, Italy; 7 U.O. Rianimazione, A.O. San Carlo Borromeo, via Pio II 3, 20147 Milan, Italy; 8 Servizio Anestesia e Rianimazione 1°, Ospedale Niguarda Ca' Granda, Piazza Ospedale Maggiore 3, 20162 Milan, Italy

## Abstract

**Introduction:**

We have recently demonstrated that metformin intoxication causes mitochondrial dysfunction in several porcine tissues, including platelets. The aim of the present work was to clarify whether it also causes mitochondrial dysfunction (and secondary lactate overproduction) in human platelets, *in vitro *and *ex vivo*.

**Methods:**

Human platelets were incubated for 72 hours with saline or increasing doses of metformin (*in vitro *experiments). Lactate production, respiratory chain complex activities (spectrophotometry), mitochondrial membrane potential (flow-cytometry after staining with JC-1) and oxygen consumption (Clark-type electrode) were then measured. Platelets were also obtained from ten patients with lactic acidosis (arterial pH 6.97 ± 0.18 and lactate 16 ± 7 mmol/L) due to accidental metformin intoxication (serum drug level 32 ± 14 mg/L) and ten healthy volunteers of similar sex and age. Respiratory chain complex activities were measured as above (*ex vivo *experiments).

**Results:**

*In vitro*, metformin dose-dependently increased lactate production (*P *< 0.001), decreased respiratory chain complex I activity (*P *= 0.009), mitochondrial membrane potential (*P *= 0.003) and oxygen consumption (*P *< 0.001) of human platelets. *Ex vivo*, platelets taken from intoxicated patients had significantly lower complex I (*P *= 0.045) and complex IV (*P *< 0.001) activity compared to controls.

**Conclusions:**

Depending on dose, metformin can cause mitochondrial dysfunction and lactate overproduction in human platelets *in vitro *and, possibly, *in vivo*.

**Trial registration:**

NCT 00942123.

## Introduction

Metformin is the drug of choice for adults with type 2 diabetes [1]. It is the seventh most frequently prescribed generic drug in the US (fifty-nine million prescriptions in 2011) [2] and is currently taken by almost two per cent of the Italian population [3].

Metformin is a safe drug [4] but lactic acidosis can develop rarely, especially when renal failure leads to accidental intoxication [5-7]. Sixty-six similar cases have been reported to the Poison Control Centre of Pavia, Italy, over the last five years, resulting in seventeen deaths (Dr. Sarah Vecchio, unpublished data). Since metformin use is constantly increasing (4% to 8% rise in prescriptions per year in the US and Italy) [2,3], related episodes of lactic acidosis will possibly become less uncommon [8].

The pathogenesis of lactic acidosis during metformin therapy remains poorly understood, particularly when no other major risk factors (such as hypoxia, tissue hypoperfusion or liver failure) can be identified [9]. Nonetheless, growing evidence suggests that metformin intoxication may directly induce lactic acidosis [10], possibly by altering liver lactate metabolism. In fact, metformin readily accumulates in hepatocytes that express the Organic Cation Transporter (OCT) 1 [11] and dose-dependently inhibits their mitochondrial respiration [12-15]. Therefore, metformin intoxication may either increase liver lactate production or decrease clearance (along with gluconeogenesis) [13,16]. The fact that OCT-1 knock-out mice do not develop lactic acidosis in response to (non severe) metformin overdose does support this model [17].

However, we have recently noted that animals [18] and humans [7] with lactic acidosis due to severe metformin overdose have a 30% to 60% decrease in their global oxygen consumption. This finding can hardly be explained solely by the inhibition of hepatic respiration. Moreover, metformin-intoxicated pigs have clear signs of mitochondrial dysfunction not just in the liver, but also in the heart, kidney, skeletal muscle and platelets [18]. Others have observed, usually *in vitro*, that metformin overdose can alter mitochondrial activity in several other tissues including animal cerebral cortex [19], pancreatic beta cells [20], neutrophils [21] and oocytes [22] and human endothelial [23], carcinoma-derived (KB) [24] and adrenal [25] cells. If extra-hepatic lactate production also globally increases (while hepatic lactate clearance decreases), then lactic acidosis will easily develop.

The aim of the present work was to clarify whether metformin intoxication alters the mitochondrial function of human platelets, taken as an example of extra-hepatic tissue. We decided to work with these cells after observing that, in pigs, metformin overdose similarly inhibits the mitochondrial activity of platelets and other more vital, but less accessible, organs [18].

## Materials and methods

The effects of metformin on human platelet mitochondria were first investigated *in vitro *and then *ex vivo*. Informed consent was always obtained. The study was approved by the Ethics Committee of the Fondazione IRCCS Ca' Granda - Ospedale Maggiore Policlinico (Milan, Italy) and registered with ClinicalTrials.gov (NCT 00942123).

### *in vitro *experiments

Platelet-rich-plasma (PRP) was obtained from whole blood of healthy donors, anticoagulated with citrate-phosphate-dextrose and then centrifuged (1000 g for 10 min). Final platelet concentration was 389 ± 55 × 10^9^/L and total leukocyte count was 24 ± 22 × 10^6^/L. It was incubated for 72 hours with saline (NaCl 154 mmol/L) or metformin (Sigma Aldrich, St. Louis, MO, USA) diluted in saline at a final concentration of 1.66 mg/L (0.01 mmol/L; therapeutic dose), 166 mg/L (1 mmol/L; toxic dose) or 16,600 mg/L (100 mmol/L; factitiously and extremely high dose), while stored at room temperature in a plastic bag permeable to air (plasma oxygen tension was always > 100 mmHg (13.3 kPa)). At the end of the incubation, plasma pH, bicarbonate, and glucose levels were measured with a blood gas analyzer (ABL 800 Flex, Radiometer GmbH, Willich, Germany) and platelets counted with a hemocytometer. Platelet respiratory chain complex activities, mitochondrial membrane potential and oxygen consumption were assessed as reported below.

In a second set of experiments, PRP was similarly incubated with saline or metformin (16,600 mg/L) but plasma pH, lactate and platelet mitochondrial membrane potential were measured every 24 hours, up to 72 hours (rather than just at 72 hours).

In a third set, PRP was incubated for 72 hours with lactic acid (30% in water) (Sigma Aldrich) or metformin (16,600 mg/L) plus sodium bicarbonate. Lactic acid was added to PRP every 24 hours so as to reach the same lactate level as the samples incubated with metformin (16,600 mg/L). Sodium bicarbonate was added every 24 hours to PRP already treated with metformin (16,600 mg/L) to maintain bicarbonate at the same level as the samples incubated with saline. Plasma pH, lactate levels and platelet oxygen consumption were measured at 72 hours.

Finally, we incubated human red blood cells, instead of platelets, with saline or metformin (16,600 mg/L) and measured pH and lactate levels every 24 hours, up to 72 hours.

### *ex vivo *experiment

We enrolled ten consecutive patients admitted since 2008 to one Hospital in Milan (Italy) with lactic acidosis (arterial pH < 7.30 and lactate concentration > 5 mmol/L), serum metformin concentration > 10 mg/L (therapeutic level is < 4 mg/L) and no other primary explanation for lactic acidosis (such as, for instance, overt respiratory, heart or liver failure). Exclusion criteria were pre-existing mitochondrial disease and hemoglobin < 8 g/dl (< 10 g/dl in the case of ischemic cardiomyopathy). Platelet mitochondrial function was studied within 48 hours of diagnosis. Blood was anticoagulated with ethylenediamine tetraacetic acid (EDTA) (30 ml) (for measuring platelet mitochondrial respiratory chain complex activities, always done) or citrate (20 ml) (for measuring platelet mitochondrial membrane potential, only performed since the beginning of 2010). It was then sedimented and centrifuged (2,500 g for 10 min) and PRP collected for further analysis (see below). Ten healthy volunteers (similar in sex and age to intoxicated patients) acted as controls.

### Mitochondrial respiratory chain complex enzyme activities

PRP (either from *in vitro *or *ex vivo *experiments) was washed with distilled water, centrifuged at 5,000 g for 10 min (14,500 g from the second cycle on) and then washed again with PBS until a clear platelet pellet could be stored at -80°C (two or three cycles were usually required). At the time of analysis, the platelet pellet was diluted in buffer (KCl 120 mM, HEPES 20 mM, MgCl_2 _5 mM and EGTA 1 mM; pH 7.2, 300 to 400 μl), sonicated (two cycles at 60 W for 10 seconds) and centrifuged (750 g for 10 min) while kept at 4°C. Supernatant was then analyzed using spectrophotometry (at 30°C). We measured the activity of respiratory chain NADH-ubiquinone 1 reductase (complex I), succinate-cytochrome c reductase (complex II+III) and cytochrome c oxidase (complex IV) and expressed it relative to that of citrate synthase (a marker of mitochondrial density) [26]. Proteins were measured using Lowry's method.

### Mitochondrial membrane potential

Platelets were diluted in plasma at 100,000/μl and kept for 30 min at 37°C in the dark with a cationic and lipophilic dye, named JC-1, that emits a green fluorescence in its native (monomeric) form. If mitochondria are normally polarized (that is, their inner milieu is negatively charged), JC-1 will accumulate into them forming dimers that emit orange, rather than green, fluorescence. This will not occur if mitochondria are not normally polarized. The ratio of normally polarized and abnormally depolarized mitochondria can then be measured as the ratio between orange and green fluorescence with flow-cytometry (JC-1 fluorescence ratio: FL2/FL1) [27].

### Platelet oxygen consumption

Platelets were resuspended in Tyrode's solution enriched with 5 mM EDTA and 1 μM prostaglandin E_1 _(final concentration 1 to 1.5 × 10^9^/ml). One ml of this suspension was transferred into a sealed chamber connected to a Clark-type electrode, and maintained at 37°C (Rank Brothers, Bottisham, UK). Oxygen consumption was recorded as the rate of decrease in oxygen tension within the chamber over the first 180 seconds (ADC-16; Pico Technology, St. Neots, UK). The results were corrected for spontaneous drift (oxygen used by the electrode itself) and platelet count (measured with a hemocytometer) [28].

### Electron microscopy

Platelets were fixed with 2.5% glutaraldehyde in PBS (pH 7.4), post-fixed in 1% osmium tetroxide and then embedded in Epon. Ultra-thin sections were counterstained with uranyl acetate and lead citrate. Mitochondrial morphology was assessed using ZEISS EM-109.

### Statistical analysis

Sample size was only calculated for experiments performed *ex vivo*. Based on preliminary *in vitro *observations, we planned to demonstrate a 30% difference in the activity of the mitochondrial respiratory chain complex I between healthy subjects and metformin-intoxicated patients (*ex vivo *experiments). Accordingly, ten individuals had to be included in each group (power 0.80 and alpha level 0.05).

Results are presented as mean and standard deviation (SD). Normally distributed data (Shapiro-Wilcoxon test) were analyzed using *t *test, one-way, one-way repeated measures or two-way repeated measures analysis of variance (ANOVA; *post-hoc *comparisons with the Holm-Sidak method). Non-normally distributed data were first transformed in ranks and then similarly analyzed. Correlation between variables was expressed as R^2 ^(linear regression analysis). A *P *value < 0.05 was considered statistically significant (SigmaPlot version 11.0, Jandel Scientific Software, San Jose, CA, USA).

## Results

**Figure 1:**
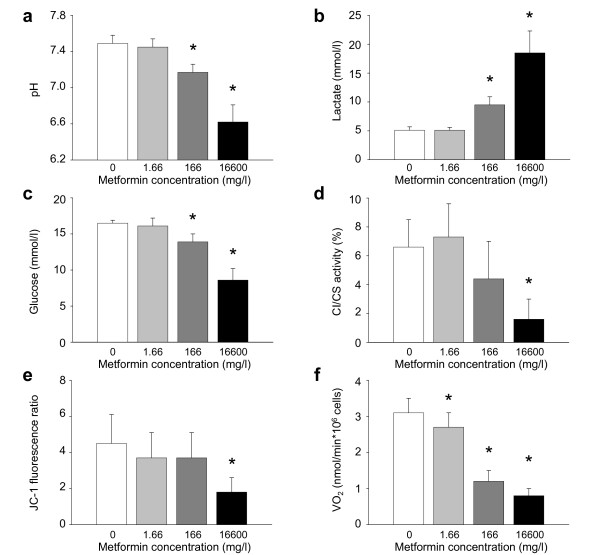
**Effects of metformin on human platelet mitochondrial function**. Platelets from healthy donors were incubated in plasma with saline (white bar) or metformin diluted in saline (concentration: 1.66 mg/L, grey bar; 166 mg/L, dark grey bar; or 16,600 mg/L, black bar). After 72 hours, (**a**) plasma pH (*P *< 0.001; one-way ANOVA), (**b**) lactate (*P *< 0.001; ANOVA on ranks) and (**c**) glucose (P < 0.001; ANOVA on ranks) concentrations, (**d**) platelet complex I (relative to citrate synthase, CI/CS) activity (*P *= 0.009; one-way ANOVA), (**e**) the proportion between normally polarized and abnormally depolarized mitochondria (JC-1 fluorescence ratio) (*P *= 0.003; one-way ANOVA) and (**f**) oxygen consumption (VO_2_) (*P *< 0.001; one-way ANOVA) were measured. Data are mean and SD, from four to eight experiments. * *P *< 0.05 versus saline (Holm-Sidak method). ANOVA, analysis of variance; SD, standard deviation.

*in vitro*, metformin increased lactate production (*P *< 0.001) and glucose consumption (*P *< 0.001), decreased respiratory chain complex I activity (*P *= 0.009), mitochondrial membrane potential (*P *= 0.003) and oxygen consumption (*P *< 0.001) of human platelets, in a dose- (Figure 1) and time-dependent [see Additional File 1] manner. Therapeutic drug dose did not alter human platelet mitochondrial function whereas toxic ones progressively did. Final plasma lactate levels inversely correlated with platelet complex I activity (R^2 ^0.54, *P *= 0.001; *n *= 16), JC-1 fluorescence ratio (R^2 ^0.37, *P *= 0.001; *n *= 32) and oxygen use (R^2 ^0.82, *P *< 0.001; *n *= 27) [see Additional File 2]. The activity of other parts of the respiratory chain and that of citrate synthase [see Additional File 3], as well as final platelet count (*P *= 0.725), did not differ between groups. Electron microscopy did not reveal major changes in platelet mitochondrial morphology.

**Figure 2:**
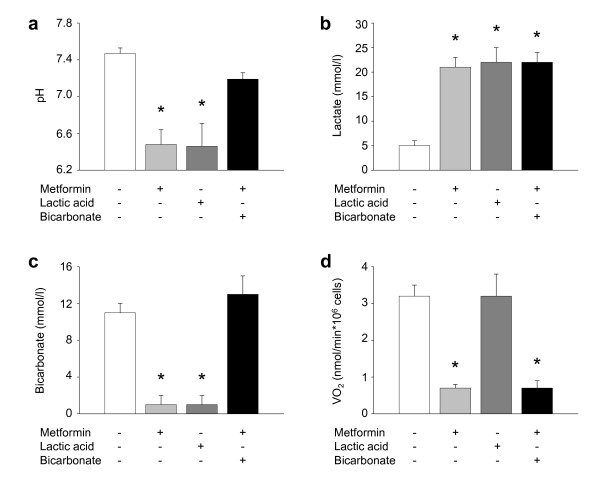
**Effects of pH on human platelet oxygen consumption**. Platelets from healthy donors were incubated in plasma with saline (white bar) or metformin diluted in saline (16,600 mg/L; grey bar), lactic acid (to mimic metformin-induced lactic acidosis; dark grey bar) or metformin diluted in saline (16600 mg/L) plus sodium bicarbonate (to correct metabolic acidosis; black bar). After 72 hours, (**a**) plasma pH (*P *< 0.001; one-way ANOVA), (**b**) lactate (*P *< 0.001; one-way ANOVA) and (**c**) bicarbonate (*P *< 0.001; one-way ANOVA) concentrations and (**d**) platelet oxygen consumption (*P *< 0.001; ANOVA on ranks) (VO_2_) were measured. Data are mean and SD, from four experiments. * *P *< 0.05 versus saline (Holm-Sidak method). ANOVA, analysis of variance; SD, standard deviation.

When lactic acid was used instead of metformin, platelet oxygen consumption never significantly diminished (despite equally severe lactic acidosis). Conversely, when sodium bicarbonate was used to mitigate metformin-induced acidosis, platelet oxygen use never returned to normal (Figure 2).

In contrast to platelets, a very high dose of metformin did not increase lactate production of human red blood cells compared to saline (*P *= 0.927) [see Additional File 4].

The effects of metformin intoxication on human platelets were also assessed *ex vivo*. Ten patients (70 ± 5 years; women 60%) with drug accumulation (serum metformin level 32 ± 14 mg/L) and lactic acidosis (arterial pH 6.97 ± 0.18 and lactate 16 ± 7 mmol/L) were enrolled. Intoxication was always accidental and associated with renal failure (creatininemia 8.9 ± 2.5 mg/dl, urea 215 ± 72 mg/dl and oligo-anuria) and continued drug intake. Possible precipitating factors were dehydration (a few days history of vomiting and diarrhea was reported in eight cases), use of potentially nephrotoxic drugs (four cases), urinary tract infection (one case) and/or complicated prostatic surgery (one case). Treatment included hemodialysis (nine cases) or continuous renal replacement therapy (one case), mechanical ventilation (two cases), catecholamines (four cases) and admission to ICU (five cases). All patients survived to hospital discharge.

**Figure 3:**
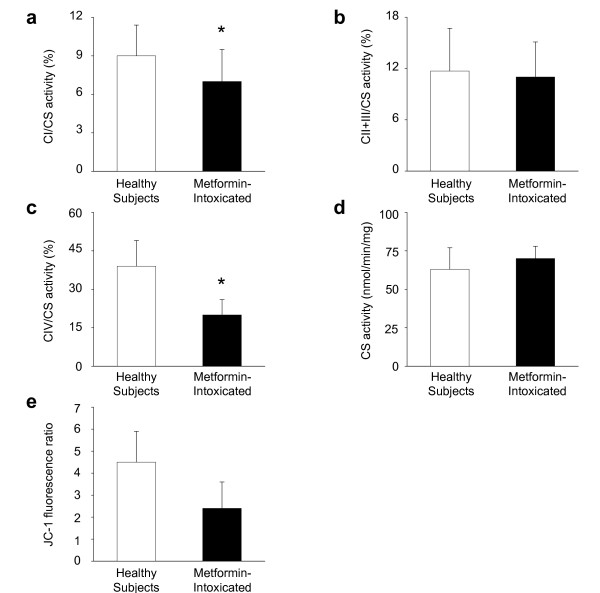
**Platelet mitochondrial function of metformin-intoxicated patients**. Ten healthy subjects (white bars) and ten metformin-intoxicated patients (black bars) were studied. The activity of platelet respiratory chain (**a**) complex I (CI) (*P *= 0.045; rank sum test), (**b**) complex II and III (CII+III) (*P *= 0.571; rank sum test) and (**c**) complex IV (CIV; *t *test) (*P *< 0.001) are expressed relative to that of (**d**) citrate synthase (CS) (*P *= 0.307; rank sum test). (**e**) The proportion between normally polarized and abnormally depolarized mitochondria was assessed in terms of JC-1 fluorescence ratio (FL2/FL1) (healthy subjects, *n *= 6; metformin-intoxicated, *n *= 4) (*P *= 0.051; *t *test). Data are mean and SD. * *P *< 0.05 versus healthy subjects;° *P *= 0.05 versus healthy subjects. SD, standard deviation.

Platelets of intoxicated patients had significantly lower complex I (*P *= 0.045) and complex IV (*P *< 0.001) activity compared to healthy controls (64 ± 9 years, women 50%) (Figure 3). The proportion between normally polarized and abnormally depolarized mitochondria, only measured in four intoxicated patients and six healthy subjects, tended to be lower in the former (*P *= 0.051) (Figure 3). Electron microscopy did not reveal any clear difference in platelet mitochondrial morphology between groups.

## Discussion

This study demonstrates that, depending on dose (and time), metformin can cause mitochondrial dysfunction and lactate overproduction in human platelets.

In fact, human platelets incubated with a high (toxic) dose of metformin had progressively lower complex I activity, mitochondrial membrane potential and oxygen consumption and higher lactate production than those incubated with saline. These changes occurred independently from hypoxia and differences in platelet count and mitochondrial density. Human platelets incubated with a low (therapeutic) dose of metformin behaved as those incubated with saline. This finding is consistent with the observation that metformin does not significantly increase the incidence of lactic acidosis, compared to other antidiabetic drugs [4], unless it accumulates.

When lactic acid was used instead of metformin to induce severe lactic acidosis, human platelet oxygen consumption never significantly declined. Conversely, when sodium bicarbonate was used to mitigate metformin-induced acidosis, human platelet oxygen consumption never returned to normal. Therefore, human platelet respiration diminishes during metformin-induced lactic acidosis because of drug accumulation, rather than (lactic) acidosis. Accordingly, healthy pigs infused with a large dose of metformin consume less oxygen than sham controls, whereas those infused with lactic acid do not (despite similar severity of lactic acidosis) [18].

When human red blood cells (that lack mitochondria) were used instead of platelets, an extremely high dose of metformin did not alter cellular metabolism. Thus, it may be concluded that metformin can cause lactate overproduction by specifically altering mitochondrial function, in human platelets as well as in mouse pancreatic ß and connective tissue cells [20,29], rat hepatocytes and skeletal muscle [12,13,30] and human intestine [31].

Aside from dose, metformin toxicity also depended on the duration of incubation. Slow drug diffusion into cells, due to inherent lipophilicity, is the most likely explanation. For this reason, patients who acutely ingest large doses of metformin may initially have very high serum drug levels but no, or only mild, lactic acidosis. In contrast, those who inadvertently get intoxicated over a few days may have relatively low serum drug levels (but still above therapeutic limits) and extremely severe lactic acidosis [6].

Our *in vitro *findings were, at least partially, replicated *ex vivo*. In fact, platelets taken from metformin-intoxicated patients had clear signs of mitochondrial dysfunction, including inhibition of complex I and IV and a lower proportion of normally polarized mitochondria (although this was only occasionally measured).

On average, patients with metformin intoxication had a 20% decrease in platelet complex I activity. We initially expected a larger effect, based on our *in vitro *experiments in which a 30%, or even larger, decrease was observed. However, the effects of metformin on mitochondria likely depend on dose, in vivo just as *in vitro*. Patients enrolled in this present study had an average serum drug level of 32 ± 14 mg/L, much lower than that of severely intoxicated patients (61 ± 25 mg/L in our previous series) [7]. *in vitro*, metformin was added to plasma to obtain an initial (toxic) concentration of 166 mg/L, or higher. Even if final drug levels were probably lower, due to cellular uptake [13], they likely exceeded 32 ± 14 mg/L. In other words, overdose severity was high (or very high) *in vitro*, but only moderate in vivo. We cannot exclude that a 24- to 48-hour delay in evaluating platelet mitochondrial function further contributed to diminish our capacity to detect early larger alterations.

The constant decrease in platelet complex IV activity was totally unexpected, as it never occurred *in vitro*, not even at the highest drug dose. Underlying mechanisms were not specifically investigated so that we can only speculate on them. Only some of the patients received sedation, catecholamines and/or mechanical ventilation, so that these factors likely had no major role. Conversely, all patients had undergone renal replacement therapy by the time their platelet mitochondrial function was assessed. Whether renal failure *per se *or extra-corporeal support can inhibit human platelet complex IV is currently unknown.

All patients enrolled in this study had a favourable outcome, despite signs of mitochondrial inhibition in platelets (and possibly other tissues). This may suggest that prognosis does not depend on the effects of metformin on the respiratory chain. However, it may also indicate that the rate of survival will be unexpectedly high if mitochondrial dysfunction is due to a compound that can be easily removed from the body (using renal replacement therapy, for instance) [32].

## Conclusions

Severe metformin overdose can alter mitochondrial function and increase lactate production of human platelets, *in vitro *and, possibly, *ex vivo*. If analogue changes also occur in other organs, they will likely contribute to the pathogenesis of metformin-induced lactic acidosis.

## Key messages

• In pigs, severe metformin intoxication causes mitochondrial dysfunction in platelets as well as in other more vital organs, including the heart, kidney and skeletal muscle.

• Human platelets exposed to a toxic dose of metformin, either *in vitro *or in vivo, have clear signs of mitochondrial dysfunction.

• If mitochondrial dysfunction is a generalized phenomenon even in humans, it will likely contribute to the development of lactic acidosis (possibly by augmenting tissue lactate production).

## Abbreviations

ANOVA: analysis of variance; EDTA: ethylenediamine tetraacetic acid; EGTA: ethylene glycol tetraacetic acid; HEPES: hydroxyl ethyl piperazine ethane sulfonic acid; NADH: nicotinamide adenine dinucleotide; OCT: organic cation transporter; PBS: phosphate buffered saline; PRP: platelet-rich-plasma; SD: standard deviation.

## Competing interests

The authors declare that they have no competing interests.

## Authors' contributions

AP conceived the study, enrolled patients, performed the analysis and wrote the manuscript. AL ran the experiments and measured mitochondrial membrane potential. FF measured respiratory chain enzyme activities and revised the manuscript. AA participated in the study design, data analysis and revised the manuscript. NG ran the experiments and helped to draft the manuscript. SV participated in data analysis and helped to draft the manuscript. GF performed electron microscopy and revised the manuscript. MM participated in study design, data analysis and revised the manuscript. GPC participated in study design, data analysis and revised the manuscript. GM participated in data analysis and helped to draft the manuscript. BL ran the experiments and revised the manuscript. LF ran the experiments and revised the manuscript. LG participated in study design, data analysis and revised the manuscript. All authors read and approved the final version of the manuscript.

## Supplementary Material

Additional File 1 **Time-dependent effects of a highly toxic dose of metformin on human platelet mitochondrial function**. Platelets from healthy donors were incubated in plasma with metformin diluted in saline (16,600 mg/L). (**a**) Plasma lactate concentration (*P *= 0.002; one-way repeated measures ANOVA) and (**b**) the ratio between normally polarized and abnormally depolarized platelet mitochondria (JC-1 fluorescence ratio) (*P *= 0.035; one-way repeated measures ANOVA) were measured every 24 hours, up to 72 hours. Data are mean and SD, from three experiments. *P < 0.05 versus time 0 (Holm-Sidak method). ANOVA, analysis of variance; SD, standard deviation. Click here for file

Additional File 2 **Relationship between platelet mitochondrial function and lactate production**. Platelets from healthy donors were incubated for 72 hours in plasma with metformin diluted in saline (concentrations ranging from 0 to 16,600 mg/L). Correlation (linear regression analysis) between final plasma lactate levels and (**a**) platelet complex I (CI) activity expressed relative to citrate synthase (CS) activity (R^2 ^0.54, *P *= 0.001; *n *= 16), (**b**) platelet JC-1 fluorescence ratio (R^2 ^0.37, *P *= 0.001; *n *= 32), and (**c**) platelet oxygen use (R^2 ^0.82, *P *< 0.001; *n *= 27) are shown. Click here for file

Additional File 3 **Dose-dependent effects of metformin on human platelet respiratory chain complex activities**. Platelets from healthy donors were incubated in plasma with saline (white bar) or metformin diluted in saline (concentration: 1.66 mg/L, grey bar; 166 mg/L, dark grey bar; or 16,600 mg/L, black bar). After 72 hours, the activity of (**a**) complex I (CI) (*P *= 0.009; one-way ANOVA), (**b**) complex II and III (CII+III) (*P *= 0.767; one-way ANOVA) and (**c**) complex IV (CIV) (*P *= 0.864; one-way ANOVA) were measured and expressed relative to that of (**d**) citrate synthase (CS) (*P *= 0.840; one-way ANOVA). Data are mean and SD from four experiments. *P < 0.05 versus saline (Holm-Sidak method). ANOVA, analysis of variance; SD, standard deviation. Click here for file

Additional File 4 **Effects of a highly toxic dose of metformin on red blood cell lactate production**. Red blood cells from healthy donors were incubated with either saline (white bar) or metformin diluted in saline (16,600 mg/L) (black bars). Lactate levels were measured every 24 hours, up to 72 hours (*P *= 0.927; two-way repeated measures ANOVA on ranks). Data are mean and SD, from three experiments. ANOVA, analysis of variance; SD, standard deviation. Click here for file

## References

[B1] Nathan DM Buse JB Davidson MB Ferrannini E Holman RR Sherwin R Zinman B American Diabetes Association, European Association for the Study of Diabetes Medical management of hyperglycaemia in type 2 diabetes: a consensus algorithm for the initiation and adjustment of therapy: a consensus statement of the American Diabetes Association and the European Association for the Study of Diabetes Diabetologia 2009 16 17 30 10.1007/s00125-008-1157-y 18941734

[B2] IMS Institute for Healthcare Informatics The use of medicines in the United States: review of 2011 http://www.imshealth.com

[B3] Gruppo lavoro OsMed L'uso dei farmaci in Italia. Rapporto nazionale Gennaio-Settembre 2011 2011 Rome

[B4] Salpeter SR Greyber E Pasternak GA Salpeter EE Risk of fatal and nonfatal lactic acidosis with metformin use in type 2 diabetes mellitus Cochrane Database Syst Rev 2010 4 CD002967 10.1002/14651858.CD002967.pub3 20091535

[B5] Peters N Jay N Barraud D Cravoisy A Nace L Bollaert PE Gibot S Metformin-associated lactic acidosis in an intensive care unit Crit Care 2008 16 R149 10.1186/cc7137 19036140 PMC2646313

[B6] Seidowsky A Nseir S Houdret N Fourrier F Metformin-associated lactic acidosis: a prognostic and therapeutic study Crit Care Med 2009 16 2191 2196 10.1097/CCM.0b013e3181a02490 19487945

[B7] Protti A Russo R Tagliabue P Vecchio S Singer M Rudiger A Foti G Rossi A Mistraletti G Gattinoni L Oxygen consumption is depressed in patients with lactic acidosis due to biguanide intoxication Crit Care 2010 16 R22 10.1186/cc8885 20170489 PMC2875537

[B8] Personne M Alarming increase of the number of metformin intoxications. Ten times doubled number of inquiries to the Swedish Poison Information Center since 2000 Lakartidningen 2009 16 994 19485031

[B9] Lalau JD Race JM Lactic acidosis in metformin therapy: searching for a link with metformin in reports of 'metformin-associated lactic acidosis' Diabetes Obes Metab 2001 16 195 201 10.1046/j.1463-1326.2001.00128.x 11412284

[B10] Wills BK Bryant SM Buckley P Seo B Can acute overdose of metformin lead to lactic acidosis? Am J Emerg Med 2010 16 857 861 10.1016/j.ajem.2009.04.012 20887905

[B11] Wang DS Kusuhara H Kato Y Jonker JW Schinkel AH Sugiyama Y Involvement of organic cation transporter 1 in hepatic and intestinal distribution of metformin J Pharmacol Exp Ther 2002 16 510 515 10.1124/jpet.102.034140 12130709

[B12] El-Mir MY Nogueira V Fontaine E Avéret N Rigoulet M Leverve X Dimethylbiguanide inhibits cell respiration via an indirect effect targeted on the respiratory chain complex I J Biol Chem 2000 16 223 228 10.1074/jbc.275.1.223 10617608

[B13] Owen MR Doran E Halestrap AP Evidence that metformin exerts its anti-diabetic effects through inhibition of complex 1 of the mitochondrial respiratory chain Biochem J 2000 16 607 614 10.1042/0264-6021:3480607 10839993 PMC1221104

[B14] Dykens JA Jamieson J Marroquin L Nadanaciva S Billis PA Will Y Biguanide-induced mitochondrial dysfunction yields increased lactate production and cytotoxicity of aerobically-poised HepG2 cells and human hepatocytes in vitro Toxicol Appl Pharmacol 2008 16 203 210 10.1016/j.taap.2008.08.013 18817800

[B15] Stephenne X Foretz M Taleux N van der Zon GC Sokal E Hue L Viollet B Guigas B Metformin activates AMP-activated protein kinase in primary human hepatocytes by decreasing cellular energy status Diabetologia 2011 16 3101 3110 10.1007/s00125-011-2311-5 21947382 PMC3210354

[B16] Fujita Y Hosokawa M Fujimoto S Mukai E Abudukadier A Obara A Ogura M Nakamura Y Toyoda K Nagashima K Seino Y Inagaki N Metformin suppresses hepatic gluconeogenesis and lowers fasting blood glucose levels through reactive nitrogen species in mice Diabetologia 2010 16 1472 1481 10.1007/s00125-010-1729-5 20349346

[B17] Wang DS Kusuhara H Kato Y Jonker JW Schinkel AH Sugiyama Y Involvement of organic cation transporter 1 in the lactic acidosis caused by metformin Mol Pharmacol 2003 16 844 848 10.1124/mol.63.4.844 12644585

[B18] Protti A Fortunato F Monti M Vecchio S Gatti S Comi GP De Giuseppe R Gattinoni L Metformin overdose, but not lactic acidosis per se, inhibits oxygen consumption in pigs Crit Care 2012 16 R75 10.1186/cc11332 22568883 PMC3580617

[B19] El-Mir MY Detaille D R-Villanueva G Delgado-Esteban M Guigas B Attia S Fontaine E Almeida A Leverve X Neuroprotective role of antidiabetic drug metformin against apoptotic cell death in primary cortical neurons J Mol Neurosci 2008 16 77 87 10.1007/s12031-007-9002-1 18040888

[B20] Hinke SA Martens GA Cai Y Finsi J Heimberg H Pipeleers D Van de Casteele M Methyl succinate antagonises biguanide-induced AMPK-activation and death of pancreatic beta-cells through restoration of mitochondrial electron transfer Br J Pharmacol 2007 16 1031 1043 17339833 10.1038/sj.bjp.0707189 PMC2013909

[B21] Zmijewski JW Lorne E Zhao X Tsuruta Y Sha Y Liu G Siegal GP Abraham E Mitochondrial respiratory complex I regulates neutrophil activation and severity of lung injury Am J Respir Crit Care Med 2008 16 168 179 10.1164/rccm.200710-1602OC 18436790 PMC2453511

[B22] Detaille D Guigas B Leverve X Wiernsperger N Devos P Obligatory role of membrane events in the regulatory effect of metformin on the respiratory chain function Biochem Pharmacol 2002 16 1259 1272 10.1016/S0006-2952(02)00858-4 11960602

[B23] Detaille D Guigas B Chauvin C Batandier C Fontaine E Wiernsperger N Leverve X Metformin prevents high-glucose-induced endothelial cell death through a mitochondrial permeability transition-dependent process Diabetes 2005 16 2179 2187 10.2337/diabetes.54.7.2179 15983220

[B24] Guigas B Detaille D Chauvin C Batandier C De Oliveira F Fontaine E Leverve X Metformin inhibits mitochondrial permeability transition and cell death: a pharmacological in vitro study Biochem J 2004 16 877 884 10.1042/BJ20040885 15175014 PMC1133963

[B25] Hirsch A Hahn D Kempná P Hofer G Nuoffer JM Mullis PE Flück CE Metformin inhibits human androgen production by regulating steroidogenic enzymes HSD3B2 and CYP17A1 and Complex I activity of the respiratory chain Endocrinology 2012 16 4354 4366 22778212 10.1210/en.2012-1145

[B26] Ragan CI Wilson MT Darley-Usmar VM Lowe PN Darley-Usmar VM, Rickwood D, Wilson MT Subfractionation of mitochondria and isolation of the proteins of oxidative phosphorylation Mitochondria: A Practical Approach 1987 Oxford: IRL Press 79 112

[B27] Verhoeven AJ Verhaar R Gouwerok EG de Korte D The mitochondrial membrane potential in human platelets: a sensitive parameter for platelet quality Transfusion 2005 16 82 89 10.1111/j.1537-2995.2005.04023.x 15647022

[B28] Protti A Carré J Frost MT Taylor V Stidwill R Rudiger A Singer M Succinate recovers mitochondrial oxygen consumption in septic rat skeletal muscle Crit Care Med 2007 16 2150 2155 10.1097/01.ccm.0000281448.00095.4d 17855829

[B29] Lenhard JM Kliewer SA Paulik MA Plunket KD Lehmann JM Weiel JE Effects of troglitazone and metformin on glucose and lipid metabolism: alterations of two distinct molecular pathways Biochem Pharmacol 1997 16 801 808 10.1016/S0006-2952(97)00229-3 9353134

[B30] Brunmair B Staniek K Gras F Scharf N Althaym A Clara R Roden M Gnaiger E Nohl H Waldhäusl W Fürnsinn C Thiazolidinediones, like metformin, inhibit respiratory complex I: a common mechanism contributing to their antidiabetic actions? Diabetes 2004 16 1052 1059 10.2337/diabetes.53.4.1052 15047621

[B31] Bailey CJ Wilcock C Scarpello JH Metformin and the intestine Diabetologia 2008 16 1552 1553 10.1007/s00125-008-1053-5 18528677

[B32] Vecchio S Protti A Metformin-induced lactic acidosis: no one left behind Crit Care 2011 16 107 10.1186/cc9404 21349142 PMC3222034

