# Supplementary material for: Metformin overdose causes platelet mitochondrial dysfunction in humans
Source: Crit Care. 2012 Oct 3;16(5):R180. doi: 10.1186/cc11663 (PMC3682281; doi:10.1186/cc11663)

Additional File 1. Time-dependent effects of a highly toxic dose of metformin on human platelet mitochondrial function. Platelets from healthy donors were incubated in plasma with metformin diluted in saline (16600 mg/l). (**a**) Plasma lactate concentration ($p=0.002$; one-way repeated measures ANOVA) and (**b**) the ratio between normally polarized and abnormally depolarized platelet mitochondria (JC-1 fluorescence ratio) ($p=0.035$; one-way repeated measures ANOVA) were measured every 24 h, up to 72 h. Data are mean and SD, from 3 experiments. *$p<0.05$ *vs.* time 0 (Holm-Sidak method).

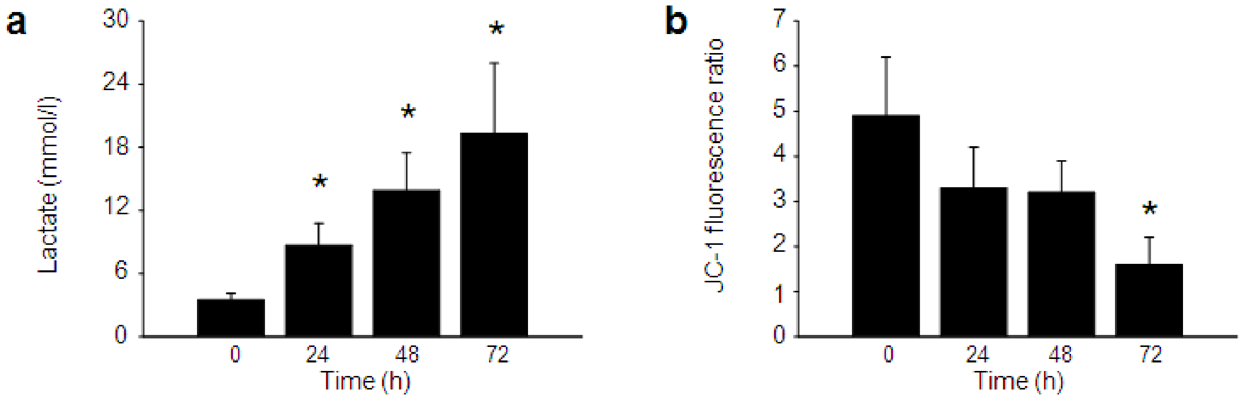

Supplement: Additional File 1 — Time-dependent effects of a highly toxic dose of metformin on human platelet mitochondrial function. Platelets from healthy donors were incubated in plasma with metformin diluted in saline (16,600 mg/L). (a) Plasma lactate concentration (P = 0.002; one-way repeated measures ANOVA) and (b) the ratio between normally polarized and abnormally depolarized platelet mitochondria (JC-1 fluorescence ratio) (P = 0.035; one-way repeated measures ANOVA) were measured every 24 hours, up to 72 hours. Data are mean and SD, from three experiments. *P < 0.05 versus time 0 (Holm-Sidak method). ANOVA, analysis of variance; SD, standard deviation. [file cc11663-S1.PDF]
