# Supplementary material for: Metformin overdose causes platelet mitochondrial dysfunction in humans
Source: Crit Care. 2012 Oct 3;16(5):R180. doi: 10.1186/cc11663 (PMC3682281; doi:10.1186/cc11663)

Additional File 2. Relationship between platelet mitochondrial function and lactate production. Platelets from healthy donors were incubated for 72 h in plasma with metformin diluted in saline (concentrations ranging from 0 to 16600 mg/l). Correlation (linear regression analysis) between final plasma lactate levels and (**a**) platelet complex I (CI) activity expressed relative citrate synthase (CS) activity (R^2 0.54, p=0.001; n=16), (**b**) platelet JC-1 fluorescence ratio (R^2 0.37, p=0.001; n=32), and (**c**) platelet oxygen use (R^2 0.82, p<0.001; n=27) are shown.

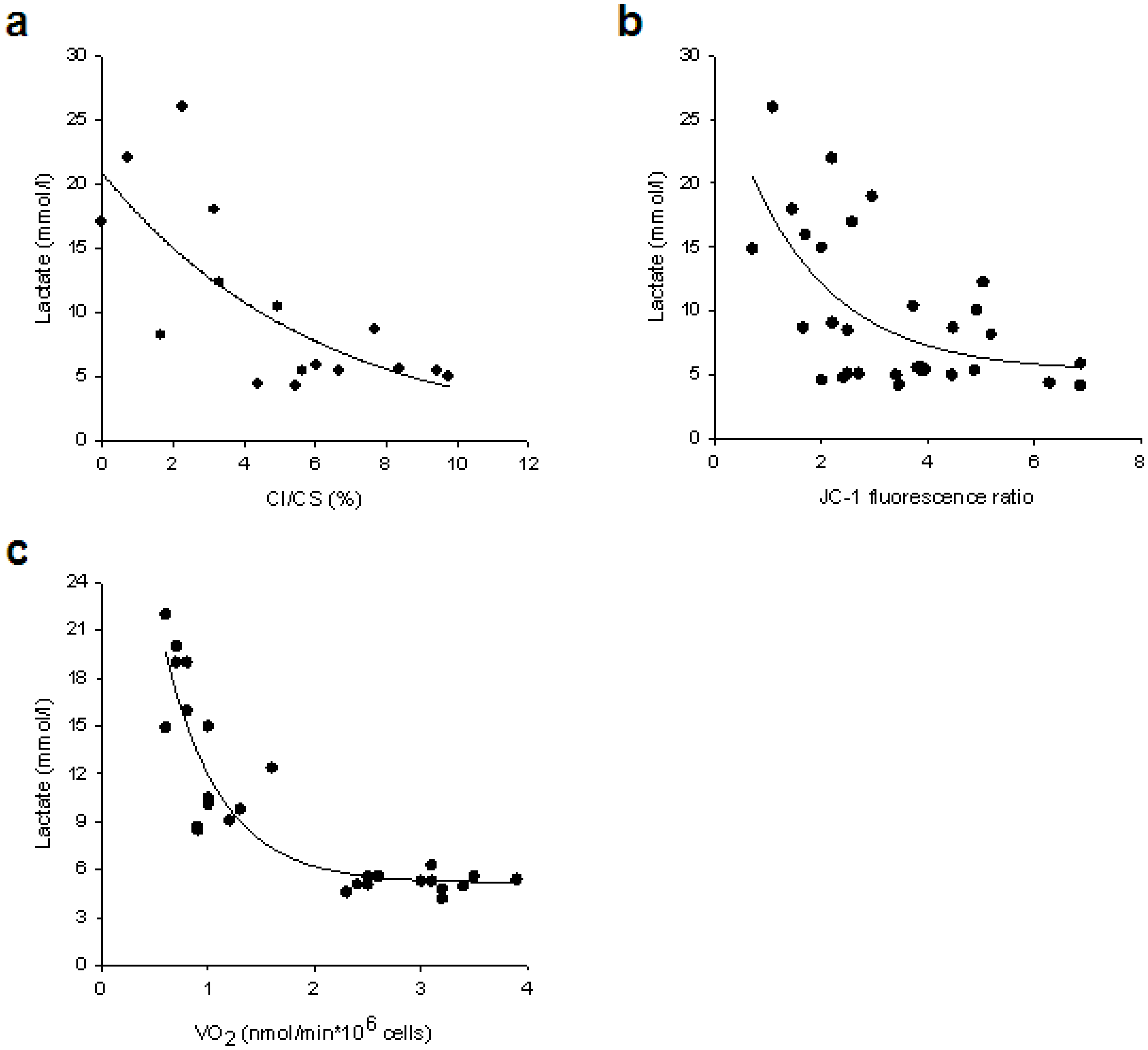

Supplement: Additional File 2 — Relationship between platelet mitochondrial function and lactate production. Platelets from healthy donors were incubated for 72 hours in plasma with metformin diluted in saline (concentrations ranging from 0 to 16,600 mg/L). Correlation (linear regression analysis) between final plasma lactate levels and (a) platelet complex I (CI) activity expressed relative to citrate synthase (CS) activity (R2 0.54, P = 0.001; n = 16), (b) platelet JC-1 fluorescence ratio (R2 0.37, P = 0.001; n = 32), and (c) platelet oxygen use (R2 0.82, P < 0.001; n = 27) are shown. [file cc11663-S2.PDF]
