# Supplementary material for: Metformin overdose causes platelet mitochondrial dysfunction in humans
Source: Crit Care. 2012 Oct 3;16(5):R180. doi: 10.1186/cc11663 (PMC3682281; doi:10.1186/cc11663)

Additional File 3. Dose-dependent effects of metformin on human platelet respiratory chain complex activities. Platelets from healthy donors were incubated in plasma with saline (white bar) or metformin diluted in saline (concentration: 1.66 mg/l, grey bar; 166 mg/l, dark grey bar or 16600 mg/l, black bar). After 72 h, the activity of (**a**) complex I (CI) ($p=0.009$; one-way ANOVA), (**b**) complex II and III (CII+III) ($p=0.767$; one-way ANOVA) and (**c**) complex IV (CIV) ($p=0.864$; one-way ANOVA) were measured and expressed relative to that of (**d**) citrate synthase (CS) ($p=0.840$; one-way ANOVA). Data are mean and SD from 4 experiments. *$p<0.05$ *vs.* saline (Holm-Sidak method).

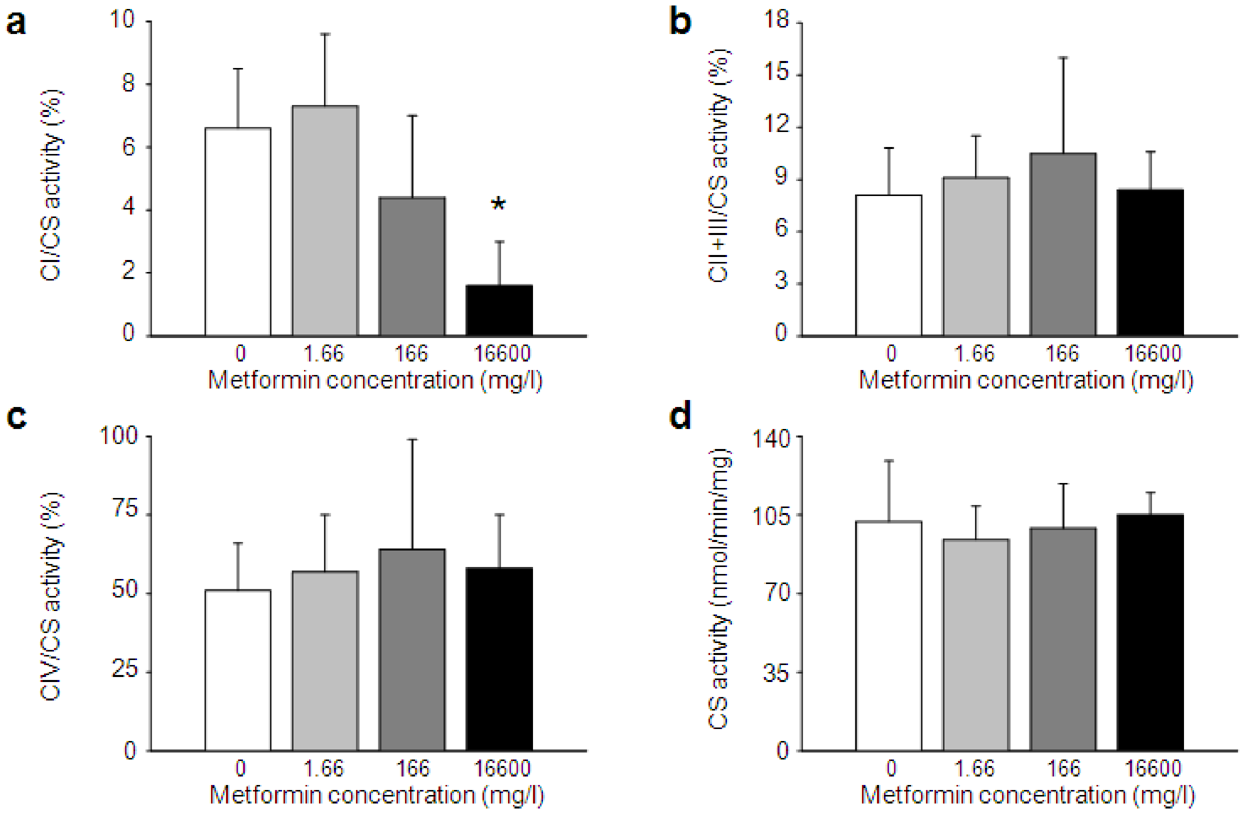

Supplement: Additional File 3 — Dose-dependent effects of metformin on human platelet respiratory chain complex activities. Platelets from healthy donors were incubated in plasma with saline (white bar) or metformin diluted in saline (concentration: 1.66 mg/L, grey bar; 166 mg/L, dark grey bar; or 16,600 mg/L, black bar). After 72 hours, the activity of (a) complex I (CI) (P = 0.009; one-way ANOVA), (b) complex II and III (CII+III) (P = 0.767; one-way ANOVA) and (c) complex IV (CIV) (P = 0.864; one-way ANOVA) were measured and expressed relative to that of (d) citrate synthase (CS) (P = 0.840; one-way ANOVA). Data are mean and SD from four experiments. *P < 0.05 versus saline (Holm-Sidak method). ANOVA, analysis of variance; SD, standard deviation. [file cc11663-S3.PDF]
