# Supplementary material for: Metformin overdose causes platelet mitochondrial dysfunction in humans
Source: Crit Care. 2012 Oct 3;16(5):R180. doi: 10.1186/cc11663 (PMC3682281; doi:10.1186/cc11663)

Additional File 4. Effects of a highly toxic dose of metformin on red blood cell lactate production. Red blood cells from healthy donors were similarly incubated with either saline (white bar) or metformin diluted in saline (16600 mg/l) (black bars). Lactate levels were measured every 24 h, up to 72 h (p=0.927; two-way repeated measures ANOVA on ranks). Data are mean and SD, from 3 experiments.

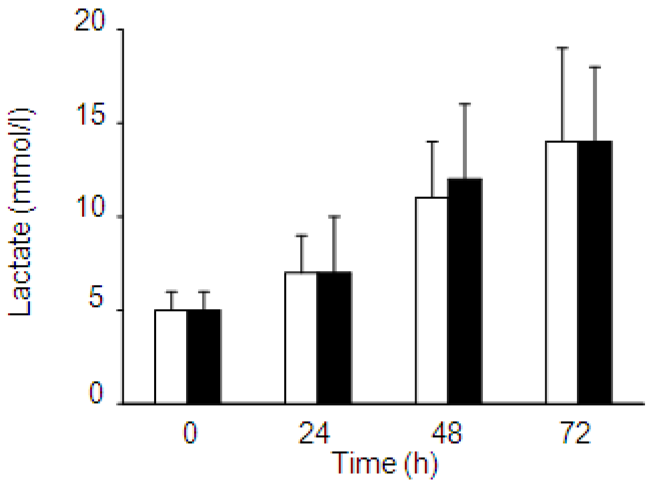

Supplement: Additional File 4 — Effects of a highly toxic dose of metformin on red blood cell lactate production. Red blood cells from healthy donors were incubated with either saline (white bar) or metformin diluted in saline (16,600 mg/L) (black bars). Lactate levels were measured every 24 hours, up to 72 hours (P = 0.927; two-way repeated measures ANOVA on ranks). Data are mean and SD, from three experiments. ANOVA, analysis of variance; SD, standard deviation. [file cc11663-S4.PDF]
